# Contributions of physically and mentally demanding working conditions to sickness presenteeism, sickness absence, and their combinations among young and early midlife municipal employees: a register-linked follow-up

**DOI:** 10.1093/annweh/wxag008

**Published:** 2026-02-19

**Authors:** Anna C Svärd, Mari-Liis Kalima, Kimmo Vänni, Eira Roos, Jatta Valkonen, Tea Lallukka

**Affiliations:** Department of Public Health, Faculty of Medicine, University of Helsinki, PO BOX 20 (Tukholmankatu 8 B), 00014 University of Helsinki, Finland; Department of Public Health, Faculty of Medicine, University of Helsinki, PO BOX 20 (Tukholmankatu 8 B), 00014 University of Helsinki, Finland; HAMK Tech, Häme University of Applied Sciences, PO BOX 230, 13101 Hämeenlinna, Finland; Department of Public Health, Faculty of Medicine, University of Helsinki, PO BOX 20 (Tukholmankatu 8 B), 00014 University of Helsinki, Finland; Department of Public Health, Faculty of Medicine, University of Helsinki, PO BOX 20 (Tukholmankatu 8 B), 00014 University of Helsinki, Finland; Department of Public Health, Faculty of Medicine, University of Helsinki, PO BOX 20 (Tukholmankatu 8 B), 00014 University of Helsinki, Finland

**Keywords:** cohort study, disability, occupational health, sick leave, workload

## Abstract

**Introduction:**

We examined how physically and mentally demanding working conditions contribute to sickness presenteeism, absence, and their combinations among young and early midlife employees.

**Methods:**

We prospectively linked the Helsinki Health Study survey on physical and mental strenuousness of work and hours per day in physical work with the employer's sickness absence register (*n* = 4,039; ages 19 to 39 in 2017). We used the Work Ability Score (WAS, work ability 0 to ­­­­10) and WAS-related discount factors to estimate annual presenteeism days lost due to reduced work ability. We calculated incidence rate ratios (IRRs) and 95% confidence intervals (CIs) for sickness presenteeism, sickness absence, and their combinations using negative binomial regression, adjusting for age, gender, marital status, and education.

**Results:**

Participants had on average 9.7 sickness presenteeism and 10.4 absence days during the mean follow-up of 0.93 yr. Overall, 57% had sickness presenteeism and 78% absence days, while 47% had both and 11% neither. Sickness presenteeism and absence days increased with the intensity of physically and mentally demanding working conditions. After age and gender adjustments, the associations were particularly strong between mentally strenuous work and sickness presenteeism (intermediate: IRR: 1.55, CI: 1.43 to 1.69; strenuous: IRR: 2.84, CI: 2.55 to 3.17), both alone or combined with absence. Adjusting for education marginally contributed to the associations concerning physical working conditions.

**Conclusions:**

The particularly strong association between mentally strenuous work and sickness presenteeism highlights the need to identify and offer targeted support to employees who perceive their work as mentally strenuous, benefiting not only individuals, but also employers and society.

What’s Important About This Paper Previous studies have shown that demanding working conditions are associated with sickness absence, but their associations with sickness presenteeism are poorly understood. This study found that young and early midlife employees with mentally and physically demanding working conditions had increased rates of sickness presenteeism and sickness absence days, which increased with the intensity of physically and mentally demanding working conditions. The findings highlight the need to identify and provide targeted support to employees who perceive their work as mentally strenuous, in addition to broader efforts to reduce mental work strain, to help prevent both sickness presenteeism and absence. These findings are especially relevant for employers and policymakers, as improving working conditions may reduce costs related to productivity loss and decreased work ability.

## Introduction

Maintaining employee health and productivity is a key challenge in modern working life, particularly in aging societies where the proportion of working-age individuals is declining, such as Japan, Italy, and Finland (OECD 2025). Traditionally, register studies have focused on measuring work ability through sickness absence or disability pension (Virtanen et al. 2020; Shiri et al. 2021). However, this captures only a part of the burden associated with work disability, as work ability may start to decrease before an actual absence from work occurs (Skagen and Collins 2016). Therefore, in recent years, there has been a growing interest in sickness presenteeism, which can simply be seen as working as ill (Aronsson et al. 2000), or from a larger perspective, as decreased productivity at work due to health problems (Schultz and Edington 2007; Ospina et al. 2015). In fact, it has been suggested that sickness presenteeism is even more costly than sickness absence (Collins et al. 2005; Garrow 2016; Kinman 2019). This especially concerns Finnish employers, who cover for the full cost of sickness presenteeism and short-term absence days, whereas the Social Insurance Institution of Finland covers for sickness absences exceeding 10 working days (Vänni et al. 2017). It is also known that sickness presenteeism is associated with sickness absence (Leineweber et al. 2012; Skagen and Collins 2016) and may occur both before and after a sickness absence period (Brouwer et al. 2002). However, the association is complex (Leineweber et al. 2012) and there is a lack of high-quality prospective studies examining sickness presenteeism, sickness absence, and their combinations, indicating an important research gap (Niedhammer et al. 2024).

Previous studies have shown that both physical and mental working conditions are associated with sickness absence (Amiri and Behnezhad 2020; Duchaine et al. 2020; Mänty et al. 2022; Sørensen et al. 2023; Sterud et al. 2024); however, we are lacking studies on their associations with sickness presenteeism. A systematic review showed that psychosocial factors at work were associated with presenteeism (Mori et al. 2021); however, almost all studies were cross-sectional. Some longitudinal studies have reported similar findings, but most originate from Japan (Oshio et al. 2017; Goto et al. 2022; Mori et al. 2023), where work culture, working conditions, and sickness absence systems differ notably from those in European countries (Brannen and Salk 2000; Dollard and Loh 2023). Additionally, the only European study focused on workplace bullying (Conway et al. 2016). There is only 1 French study examining both physical and mental working conditions and their associations with sickness presenteeism alone or combined with sickness absence, based on a national sample of 16,129 French employees (Niedhammer et al. 2024). The study found that both psychosocial and physical work factors were associated with sickness presenteeism, and that working conditions were even more strongly associated with sickness presenteeism than with sickness absence alone. Information on working conditions was derived from 2013, whereas sickness absence data were self-reported in 2016 in that study.

Mental disorders and musculoskeletal diseases are the major causes of sickness absence in Finland, accounting for more than 60% of all long-term sickness absence days (The Social Insurance Institution of Finland 2025). Among Finns under the age of 40, sickness absence has increased rapidly, especially due to mental disorders, which has raised concerns about the work ability of young employees (Blomgren and Perhoniemi 2022). Therefore, it is important to increase the understanding of the associations between physical and mental working conditions and both sickness presenteeism and sickness absence especially among young and early midlife employees who still have most of their working life ahead. As employers are responsible for organizational performance and the costs associated with sickness presenteeism, they are likely to be interested in identifying and implementing preventive measures to reduce it. As social and socioeconomic factors are associated with both working conditions and work ability (Pekkala et al. 2017; Sutela et al. 2024), we included these as covariates.

The aim of this register-linked follow-up study was to examine the associations of physical and mental working conditions with sickness presenteeism, sickness absence, and their combinations among young and early midlife employees, controlling for key covariates. Based on previous research, we assumed that both physical and mental working conditions are expected to be associated with sickness absence (Amiri and Behnezhad 2020; Duchaine et al. 2020; Mänty et al. 2022; Sørensen et al. 2023; Sterud et al. 2024) and sickness presenteeism (Mori et al. 2021). However, a previous French study suggested that these associations may differ when sickness absence and sickness presenteeism are examined jointly (Niedhammer et al. 2024).

## Methods

### Participants

**Figure 1:**
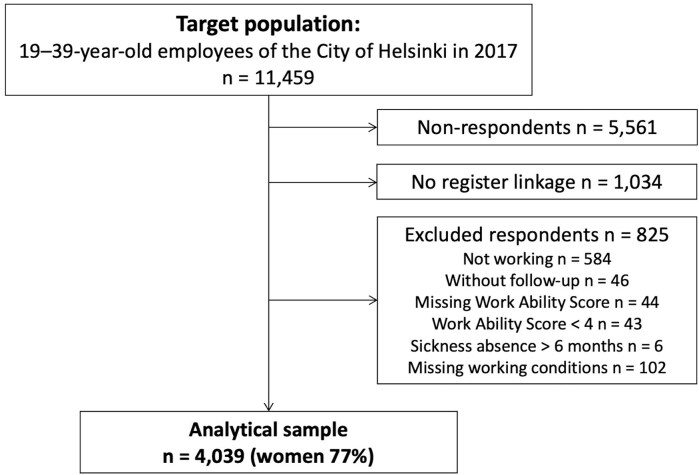
Flow chart of the study population selection.

The Helsinki Health Study is an ongoing cohort study on employees of the City of Helsinki, which is the largest employer in Finland with around 38,000 employees representing a wide range of occupations and sectors (Lallukka et al. 2020). Data were collected via online and postal questionnaires among 19- to 39-yr-old employees with at least a 50% employment contract for a minimum of 4 mo before the start of the data collection in 2017 (target population *n* = 11,459). Among those who did not respond to the online or postal questionnaire, a shorter telephone interview (*n* = 787) was also conducted. In total, 5,898 participants responded (response rate 51.5%), and of these, 4,864 (82%) consented to register linkage (Fig. 1). The majority of respondents were women (78%), which reflects the gender distribution of the target population and the municipal sector in Finland (Lallukka et al. 2020). According to the nonresponse analysis, the respondents broadly represented the target population, although those with a lower socioeconomic position and any long-term sickness absence both before and during the data collection were somewhat less likely to respond (Lallukka et al. 2020).

We excluded participants who were not working full- or part-time at baseline (*n* = 584), who had a sickness absence lasting more than 6 mo during the follow-up (*n* = 6), those who were without follow-up data (*n* = 46), and who had missing information on Work Ability Score (WAS) (*n* = 44) or working conditions (*n* = 102). As in a previous study (Vänni et al. 2017), we excluded participants with a WAS below 4 (*n* = 43), because those with a WAS of 0 to 3 tend to have high levels of sickness absence, which may lead to overestimated levels of presenteeism. The final dataset used in the analyses consisted of 3,091 women and 948 men.

### Exposure: working conditions

We used 3 self-reported measures of demanding working conditions: physical and mental strenuousness of work and hours per day spent in heavy physical work. Physical and mental strenuousness of work were asked for in the questionnaire with 4 response options ranging from very light to very strenuous (Laaksonen et al. 2010; Lahti et al. 2021).

We categorized physically strenuous work into 3 groups: (i) strenuous (rather/very strenuous), (ii) intermediate (rather light), and (iii) nonstrenuous (very light), because employees with physically very strenuous work were few (*n* = 150) (Lahti et al. 2021). Mentally strenuous work was categorized as follows: (i) strenuous (very strenuous), (ii) intermediate (rather strenuous), and (iii) nonstrenuous (rather/very light), because employees with mentally very light work were few (*n* = 39). It is possible that people with poorer health perceive their work as physically more strenuous, and therefore, we also used a more objective measure on physically demanding working conditions. In the measure, participants estimated how many hours per day they do physically strenuous work, such as heavy lifting and climbing stairs. We categorized participants as follows: (i) those who did no physical work, (ii) those who spent 2 h or less per day in physical work, and (iii) those who spent more than 2 h per day in heavy physical work.

### Outcome measures: sickness presenteeism, sickness absence, and their combinations

We used information on sickness absence from the personnel register of the City of Helsinki. The follow-up of sickness absence started the day after we received the questionnaire from a participant and continued for 1 yr or until the end of the participant's employment contract, whichever came first (mean follow-up of 0.93 yr). We calculated the total number of sickness absence days during the follow-up year, including also self-certified sickness absence days, which in certain circumstances can be authorized by supervisors without a medical certificate.

We used a presenteeism measurement tool developed and validated by Vänni et al. to approximate the number of annual presenteeism days lost due to reduced work ability using register based information on sickness absence days and survey information on WAS, which is a person's estimate of one's current work ability on a scale from 0 to 10, compared to one's lifetime best (Vänni et al. 2018b). It has been shown that, in theory, a 1-point decrease below 9 on the WAS is associated with an approximately 5% decrease in productivity (Vänni 2018a). First, we calculated annual net working days by subtracting annual sickness absence days from the annual working days, which was estimated to 227 days, according to the average number of working days in Finland (Confederation of Finnish Industries 2022). Next, we adjusted the net working days based on the individual's follow-up time and self-reported weekly working hours (38.25 h used as a standard weekly working time at the City of Helsinki). For those with missing information on working hours (*n* = 553, 87% of whom were phone respondents), we used the standard weekly working hours (38.25 h). Finally, we multiplied the annual net working days with WAS-related discount factors to get an estimate of annual number of presenteeism days.

We examined sickness absence and sickness presenteeism days separately, but we also conducted analyses in which we examined sickness absence and sickness presenteeism combined. In these combined analyses we distinguished between participants with neither sickness absence nor sickness presenteeism, those with sickness absence only, those with sickness presenteeism only, and those with both outcomes during the follow-up year.

### Covariates

Covariates were derived from the survey. We used 3 age groups: 19 to 29, 30 to 34, and 35 to 39 yr. We classified gender as women and men, marital status as married/cohabiting and others (single, divorced, or widowed), and education as upper secondary school or less, bachelor's degree, and master's degree or higher.

Participants with missing information on marital status (*n* = 8) or education (*n* = 7) were few, and to avoid unnecessary cumulative exclusions, they were classified as single and having low education, respectively (Svärd et al. 2025).

### Statistical analyses

First, we used cross-tabulation to describe the distribution of characteristics among the participants at baseline according to sickness presenteeism and sickness absence days combined. Second, we used negative binomial regression to calculate the incidence and 95% confidence intervals (CIs) for sickness presenteeism and sickness absence days per 10 person-years by exposure to demanding working conditions. Third, we calculated incidence rate ratios (IRRs) and 95% CIs for sickness presenteeism and sickness absence days using negative binomial regression models. Participants without exposure to demanding working conditions served as the reference group. Model 1 was adjusted for age and gender, and Model 2 additionally for marital status and education. We included a natural logarithm of the follow-up time as an offset variable in the models to account for different follow-up lengths. Finally, we calculated relative risk ratios (RRRs) and 95% CIs for sickness presenteeism and sickness absence days combined using multinomial logistic regression models. Participants without exposure to demanding working conditions and without sickness presenteeism and sickness absence days served as reference groups.

To examine whether we could analyze women and men together, we conducted gender-interaction tests. There were no statistically significant gender interactions for physically strenuous work (*P* = 0.144 for sickness presenteeism and *P* = 0.546 for sickness absence), nor for hours per day spent in heavy physical work (*P* = 0.089 for sickness presenteeism and *P* = 0.983 for sickness absence). However, for mentally strenuous work, the interaction was significant for both sickness presenteeism (*P* = 0.038) and sickness absence (*P* = 0.015), even though the results were mainly similar. Due to the low number of men, we pooled women and men in the analyses and present gender-stratified analyses for mentally strenuous work as a supplement (Tables S1 and S2).

We used IBM SPSS Statistics 29 for the analyses.

## Results

During the follow-up year (mean follow-up 0.93 yr), participants had on average 9.7 sickness presenteeism and 10.4 sickness absence days. Overall, 57% of participants had sickness presenteeism days and 78% had sickness absence days, while 47% had both and 11% had neither (Table 1).

**Table 1:** Characteristics of the young and early midlife employees of the City of Helsinki, Finland, at Phase 1 in 2017, by sickness presenteeism and sickness absence days during the 1-yr follow-up.

	All	No presenteeism days		Presenteeism ≥1 d		
		No sickness absence days	Sickness absence ≥1 day	No sickness absence days	Sickness absence ≥1 day	
Characteristics at Phase 1	*n* (%)	*n* (%)	*n* (%)	*n* (%)	*n* (%)	*P*-value^a^
*Total n (%)*	4,039 (100)	454 (11)	1,265 (31)	425 (11)	1,895 (47)	…
*Age*						
18 to 29	1,294 (32)	136 (11)	432 (33)	115 (9)	611 (47)	0.096
30 to 34	1,325 (33)	164 (12)	404 (30)	153 (12)	604 (46)	…
35 to 39	1,420 (35)	154 (11)	429 (30)	157 (11)	680 (48)	…
*Gender*						
Women	3,091 (23)	285 (9)	968 (31)	302 (10)	1,536 (50)	<0.001
Men	948 (77)	169 (20)	297 (31)	123 (13)	359 (38)	…
*Marital status*						
Married/cohabiting	2,624 (65)	305 (12)	848 (32)	279 (11)	1,192 (45)	0.072
Others	1,415 (35)	149 (11)	417 (29)	146 (10)	703 (50)	…
*Education*						
Upper secondary school	1,386 (34)	105 (8)	448 (32)	118 (9)	715 (52)	<0.001
Bachelor's degree	1,439 (36)	174 (12)	439 (31)	143 (10)	683 (47)	…
Master's degree or higher	1,214 (30)	175 (14)	378 (31)	164 (14)	497 (41)	
*Physically strenuous work*						
Nonstrenuous	883 (22)	137 (15)	299 (34)	104 (12)	343 (39)	<0.001
Intermediate	1,793 (44)	198 (11)	585 (33)	200 (12)	810 (45)	…
Strenuous	1,363 (34)	119 (9)	381 (28)	121 (9)	742 (54)	…
*Mentally strenuous work*						
Nonstrenuous	897 (22)	144 (16)	385 (43)	77 (9)	291 (32)	<0.001
Intermediate	2,501 (62)	280 (11)	772 (31)	273 (11)	1,176 (47)	…
Strenuous	641 (16)	30 (5)	108 (17)	75 (12)	428 (67)	…
*Time spent in physical work*						
0 h	1,447 (36)	201 (14)	453 (31)	165 (11)	628 (43)	<0.001
>0 to 2 h	1,508 (37)	163 (11)	469 (31)	172 (11)	704 (47)	…
>2 h	1,084 (27)	90 (8)	343 (32)	88 (8)	563 (52)	…

^a^ *P*-values from chi-squared tests.

**Figure 2:**
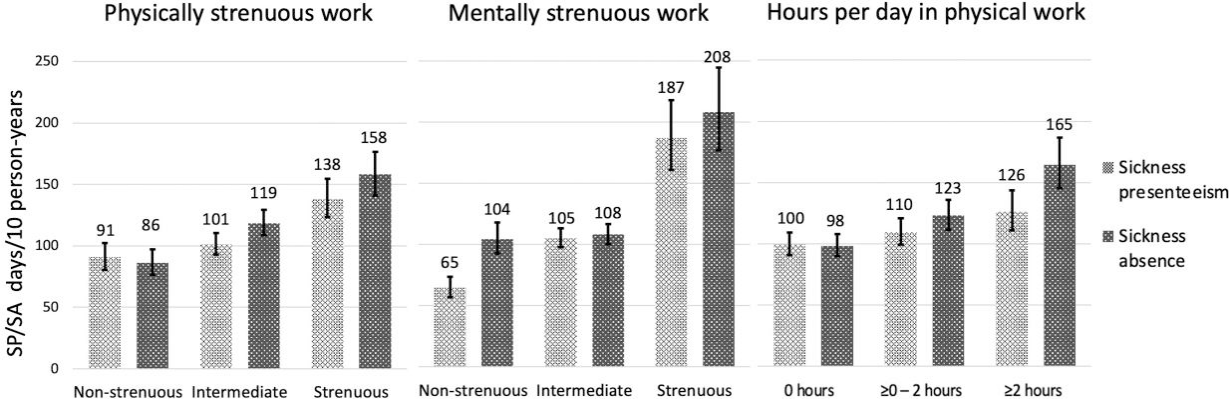
Age- and gender-adjusted incidence of sickness presenteeism (SP) and sickness absence (SA) days during a 1-yr follow-up with 95% CIs per 10 person-years according to exposure to demanding working conditions among young and early midlife employees of the City of Helsinki, Finland in 2017 (*n* = 4,039).

Figure 2 presents age- and gender-adjusted incidences of sickness presenteeism and sickness absence days per 10 person-years by exposure to demanding working conditions. Participants with exposure to physically and mentally demanding working conditions had a higher incidence of both sickness presenteeism and sickness absence days, suggesting that the associations become stronger with increasing work demands, particularly for mentally strenuous work.

Similar associations between demanding working conditions and sickness absence were found based on negative binomial regression estimates (IRRs) (Table 2). Overall, the associations between physically demanding working conditions and sickness presenteeism were weaker than for sickness absence, especially after adjusting for marital status and education, which attenuated the association for hours per day in heavy physical work (>0 to 2 h: IRR 1.03, 95% CI 0.95 to 1.11; >2 h: IRR 1.07, 95% CI 0.97­ to 1.17), but not for physically strenuous work. However, the associations for mentally strenuous work remained strong (intermediate: IRR 1.61, 95% CI 1.48 to ­1.75; strenuous: IRR 2.97, 95% CI 2.67 to 3.32). The unadjusted gender-stratified results suggested weaker associations between mentally strenuous work and sickness absence among men than women, but the associations were otherwise similar (Tables S1 and S2).

**Table 2:** IRRs and their 95% CIs for sickness presenteeism days and sickness absence days during a 1-yr follow-up among young and early midlife employees of the City of Helsinki, Finland (*n* = 4,039), by exposure to demanding working conditions in 2017.

		Sickness presenteeism days				Sickness absence days			
		Model 1		Model 2		Model 1		Model 2	
Exposure group	*n* (%)	IRR	95% CI	IRR	95% CI	IRR	95% CI	IRR	95% CI
*Physically strenuous work*									
Nonstrenuous	883 (22)	1.00	…	1.00	…	1.00	…	1.00	…
Intermediate	1,793 (44)	1.11	1.02 to 1.21	1.10	1.01 to 1.20	1.30	1.19 to 1.42	1.20	1.10 to 1.31
Strenuous	1,363 (34)	1.32	1.21 to 1.45	1.28	1.16 to 1.41	1.63	1.48 to 1.78	1.27	1.15 to 1.40
*Mentally strenuous work*									
Nonstrenuous	897 (22)	1.00	…	1.00	…	1.00	…	1.00	…
Intermediate	2,501 (62)	1.55	1.43 to 1.69	1.61	1.48 to 1.75	1.13	1.04 to 1.23	1.23	1.13 to 1.36
Strenuous	641 (16)	2.84	2.55 to 3.17	2.97	2.67 to 3.32	1.67	1.50 to 1.86	1.87	1.67 to 2.08
*Time spent in physical work per day*									
0 h	1,447 (36)	1.00	…	1.00	…	1.00	…	1.00	…
>0 to 2 h	1,508 (37)	1.05	0.97 to 1.13	1.03	0.95 to 1.11	1.23	1.14 to 1.33	1.12	1.03 to 1.21
>2 h	1,084 (27)	1.14	1.05 to 1.24	1.07	0.97 to 1.17	1.61	1.48 to 1.75	1.26	1.15 to 1.38

Negative binomial regression.

Model 1: Adjusted for age and gender.

Model 2: Adjusted for Model 1 + marital status and education.

When examining the associations between demanding working conditions and sickness presenteeism and sickness absence days—alone or combined—using multinominal logistic regression, physically strenuous work was associated with sickness presenteeism and sickness absence days combined (intermediate: RRR: 1.66, 95% CI: 1.29 to 2.14; strenuous: RRR 2.43, 95% CI 1.83 to 3.22) and with sickness absence days only (intermediate: RRR: 1.35, 95% CI: 1.04 to 1.76; strenuous: RRR 1.39, 95% CI 1.04 to 1.89) (Table 3). The associations with sickness presenteeism only were weaker (intermediate: RRR: 1.35, 95% CI: 0.98 to 1.87; strenuous: RRR 1.36, 95% CI 0.95 to 1.96). The results were similar for hours per day spent in heavy physical work. However, for mentally strenuous work, the association for sickness absence only was weak, but strong for sickness presenteeism only (intermediate: RRR 1.76, 95% CI 1.27 to ­2.44; strenuous: RRR 4.59, 95% CI 2.77 to 7.63) and for sickness presenteeism and sickness absence combined (intermediate: RRR 1.89, 95% CI: 1.48 to ­2.41; strenuous: RRR 6.63, 95% CI 4.34 to 10.13).

**Table 3:** Age- and gender-adjusted relative risk ratios (RRRs) and their 95% confidence intervals (CIs) for sickness presenteeism days, sickness absence days, and their combinations during a 1-yr follow-up among young and early midlife employees of the City of Helsinki, Finland (*n* = 4,039), by exposure to demanding working conditions in 2017.

		No presenteeism days				Presenteeism ≥1 d			
		No sickness absence days (ref.)		Sickness absence ≥1 d		No sickness absence days		Sickness absence ≥1 d	
Exposure group	*n* (%)	RRR	CI	RRR	CI	RRR	CI	RRR	CI
*Physically strenuous work*									
Intermediate	1,895 (44)	1.00	—	1.35	1.04 to 1.76	1.35	0.98 to 1.87	1.66	1.29 to 2.14
Strenuous	1,363 (34)	1.00	—	1.39	1.04 to 1.89	1.36	0.95 to 1.96	2.43	1.83 to 3.22
*Mentally strenuous work*									
Intermediate	2,501 (62)	1.00	—	0.95	0.75 to 1.21	1.76	1.27 to 2.44	1.89	1.48 to 2.41
Strenuous	641 (16)	1.00	—	1.27	0.81 to 1.99	4.59	2.77 to 7.63	6.63	4.34 to 10.13
*Time spent in physical work per day*									
>0 to 2 h	1,508 (37)	1.00	—	1.31	1.03 to 1.68	1.33	0.99 to 1.80	1.45	1.15 to 1.84
>2 h	1,084 (27)	1.00	—	1.75	1.31 to 2.34	1.27	0.88 to 1.83	2.16	1.63 to 2.85

Multinomial logistic regression.

Reference categories were physically nonstrenuous work (*n* = 883), mentally nonstrenuous work (*n* = 897), and 0 h spent in physical work (*n* = 1,447).

## Discussion

Our study showed that the rates of both sickness presenteeism and sickness absence days increased with the intensity of physically and mentally demanding working conditions among young and early midlife employees. The associations were particularly strong between mentally strenuous work and sickness presenteeism, both alone and combined with sickness absence, whereas no statistically significant associations were found between mentally strenuous work and sickness absence only. The associations between physically strenuous work and sickness absence were weaker, and no associations were found between physically demanding working conditions and sickness presenteeism only.

Our results are in line with previous studies, which have shown that demanding working conditions are associated with sickness presenteeism (Mori et al. 2021; Niedhammer et al. 2024) and sickness absence (Amiri and Behnezhad 2020; Duchaine et al. 2020; Mänty et al. 2022; Sørensen et al. 2023; Sterud et al. 2024). However, previous longitudinal research on sickness presenteeism is limited and has focused on psychosocial factors only (Conway et al. 2016; Oshio et al. 2017; Goto et al. 2022; Mori et al. 2023), even though physical working conditions, which particularly increase musculoskeletal strain and remain common among employees with lower socioeconomic position (Murtin et al. 2022), are known to be associated with sickness absence among young employees (Mänty et al. 2022). Additionally, most of the previous studies were from Japan, where work culture, working conditions, and sickness absence systems are different from those in European countries (Brannen and Salk 2000; Dollard and Loh 2023).

To the best of our knowledge, only 1 previous French study has examined the associations of physical and mental working conditions with sickness presenteeism and sickness absence combined, among 16,129 French employees aged 15 to 65 yr followed from 2013 to 2016 (Niedhammer et al. 2024). In line with the French study, our study showed that the association between mentally strenuous work and sickness presenteeism only or combined with sickness absence was strong, but statistically nonsignificant for sickness absence only. In contrast, physically demanding working conditions were associated with sickness absence but not with sickness presenteeism only in our study. Employees with mentally demanding work may have the opportunity to work from home, for example when suffering from the flu, in contrast to those with physically demanding work. Additionally, the threshold for taking sick leave may be higher if the work is difficult to substitute and taking sick leave would mean that no one will handle the tasks in the meantime.

Our findings are consistent with a previous study using the same cohort, which reported a dose–response association between physical working conditions and sickness absence (Mänty et al. 2022). In the French study, the association with sickness presenteeism only was strong for men, but weaker and only borderline significant for women. The number of men was low in our study, but we did gender-stratified sensitivity analyses and the results remained similar between genders (Tables S1 and S2). The French study used median-based cutoffs and did not account for intensity of the physical exposures, as we did. Our population was younger, which may also explain the differences: in Finland, mental ill-health is the leading cause of sickness absence among young workers, whereas musculoskeletal disorders increase with age (Blomgren and Perhoniemi 2022; The Social Insurance Institution of Finland 2025). One could speculate that older employees have chronic health problems, such as musculoskeletal disorders and physical work limitations, but continue working despite these, which may increase presenteeism among this age group.

We used register-based data for sickness absence and estimated sickness presenteeism days using WAS, whereas in the French study, the annual number of sickness presenteeism and absence days were self-reported 3 yr after the initial survey. WAS is associated with both physical and mental health and reflects the holistic view that work ability is more than health alone, as it takes into account the balance between individual resources (health, competence, values and motivation) and work-related factors within the organizational and societal context, illustrated by the “House of Work Ability” (Gould et al. 2008). Having both sickness presenteeism and absence (47% vs. 21%) or absence only (31% vs. 13%) was more common in our study, while presenteeism only (11% vs. 26%) or neither (11% vs. 39%) was more common in the French study. Despite methodological differences and a higher proportion of women in our study (among whom presenteeism is more common), overall presenteeism rates were quite similar (57% vs. 47%). However, thresholds for both sickness presenteeism and sickness absence may vary across populations and age groups, highlighting the need for further studies in diverse cultural and demographic contexts.

Given that both sickness presenteeism and sickness absence days were common among the young and early midlife employees, these findings highlight the importance of supporting work ability in this age group—not only for individual health, but also from a societal perspective. Maintaining the work ability of younger employees is essential for the sustainability of social and economic systems in the context of an aging population (1).

Our findings are also highly relevant for employers, especially in Finland, where employers are responsible for the full costs of sickness presenteeism and short-term absences. There is a clear need to identify effective preventive strategies in the workplace and to conduct further research that takes into account the economic burden of both sickness presenteeism and sickness absence.

### Methodological considerations

There are some limitations that should be noted. First, the response rate in the survey was acceptable (51.5%), but nonresponse and risk of a healthy worker effect remain a potential limitation, even though it is shown that the respondents in this study represent the target population broadly (Lallukka et al. 2020), and we were able to improve the socioeconomic representativeness of the data by including those who also responded to the shorter telephone interview. However, individuals with lower socioeconomic position and those with long-term sickness absence were less likely to respond, and it is possible that, for example, employees with a physically demanding job are underrepresented.

Second, information on working conditions and covariates was self-reported, and participants with poorer health might be more likely to perceive their job as strenuous, which can lead to reverse causation. However, objective data on working conditions are rarely available and typically reflects group-level exposure, which does not capture individual perceptions that may vary even within the same job. We measured working conditions with single-item measures. However, a previous study has shown that the single-item measure of mentally strenuous work used in this study correlates with Karasek's model of job demands and job strain, although not with job control (Lahti et al. 2021). Similarly, the single-item measures of physical working conditions correlate with multi-item measures of physical work and hazardous exposures and have shown similar associations with sickness absence (Lahti et al. 2021; Svärd et al. 2025).

Third, although sickness absence was based on reliable register data, sickness presenteeism was estimated indirectly using a presenteeism measurement tool developed and validated by Vänni et al. (Vänni et al. 2018b). The tool is based on the WAS, and its validity has been confirmed in 2 Finnish industrial sectors (food and forest industry) (Vänni 2018a). Each 1-point decrease below 9 on the WAS is theoretically associated with an approximate 5% decrease in work productivity, which, combined with sickness absence data, allows the estimation of annual presenteeism days. The method has not been validated in the public sector, but it lies on the logical assumption: when perceived work ability declines, so does an individual's capacity to maintain full productivity while continuing to work. This makes the scale a practical proxy for measuring productivity loss due to presenteeism, especially in the absence of direct productivity measures.

Fourth, although the cohort included a wide range of occupations and social classes, the generalizability of the findings is limited, as it consisted of Finnish public sector employees, most of whom live in the capital region. Differences in work culture and employment security between countries and workplaces may limit the generalizability of the findings and highlight the importance of further studies in different settings. Additionally, in line with the gender distribution in the public sector more broadly, most participants were women, which may limit the ability to draw gender-specific conclusions. Nevertheless, the use of a large cohort combined with reliable register-based data enabled us to provide important new insights into the associations between working conditions and both sickness absence and sickness presenteeism among young and early midlife employees, whose ability to remain healthy and employed is increasingly important, especially in aging societies with increasing demands on the labor force.

## Conclusions

Physically and mentally demanding working conditions were associated with both sickness presenteeism and sickness absence days, which were both common among the young and early midlife employees and often co-occurred. The particularly strong association between mentally strenuous work and sickness presenteeism—both alone and in combination with sickness absence—highlights the need to identify employees who perceive their work as mentally strenuous. Targeted support for these employees, together with broader efforts to reduce mental work strain, may help prevent productivity loss and promote employee well-being. These measures could benefit not only individuals, but also employers and society at large.

## Supplementary Material

wxag008_Supplementary_Data

## Data Availability

Data are not publicly available due to strict data protection laws and can only be accessed by the members of the study group (https://www.helsinki.fi/en/researchgroups/helsinki-health-study/data-protection-statement). Further inquiries can be directed to the corresponding author.
