# Supplementary material for: Contributions of physically and mentally demanding working conditions to sickness presenteeism, sickness absence, and their combinations among young and early midlife municipal employees: a register-linked follow-up
Source: Ann Work Expo Health. 2026 Feb 19;70(2):wxag008. doi: 10.1093/annweh/wxag008 (PMC13017116; doi:10.1093/annweh/wxag008)
Supplement: wxag008_Supplementary_Data [file wxag008_supplementary_data.pdf]

SUPPLEMENTARY DATA

Title: Contributions of physically and mentally demanding working conditions to sickness presenteeism, sickness absence, and their combinations among young and early midlife municipal employees: a register-linked follow-up

Authors: Anna C. Svärd[1], Mari-Liis Kalima[1], Kimmo Vänni[2], Eira Roos[1], Jatta Valkonen[1], Tea Lallukka[1]

Affiliations: [1]Department of Public Health, Faculty of Medicine, University of Helsinki, Helsinki, Finland; [2]HAMK Tech, Häme University of Applied Sciences, Hämeenlinna, Finland

Supplemental Table S1. Gender-stratified incidence rate ratios (IRRs) and their 95% confidence intervals (CIs) for sickness presenteeism days, sickness absence days, and their combinations during a 1-year follow-up among young and early midlife employees of the City of Helsinki, Finland (n = 4,039), by exposure to mentally strenuous work in 2017. Negative binomial regression.

			Sickness presenteeism days				Sickness absence days			
			Model 1		Model 2		Model 1		Model 2	
	Exposure group	n (%)	IRR	95% CI	IRR	95% CI	IRR	95% CI	IRR	95% CI
Women	*Mentally strenuous work*									
	Non-strenuous	608 (20)	1.00		1.00		1.00		1.00	
	Intermediate	1983 (64)	1.65	1.50–1.83	1.69	1.53–1.87	1.20	1.09–1.32	1.26	1.15–1.39
	Strenuous	500 (16)	2.96	2.61–3.36	3.06	2.69–3.47	1.82	1.61–2.06	1.96	1.72–2.22
Men	*Mentally strenuous work*									
	Non-strenuous	289 (31)	1.00		1.00		1.00		1.00	
	Intermediate	518 (55)	1.32	1.13–1.54	1.43	1.22–1.68	0.98	0.84–1.14	1.18	1.01–1.39
	Strenuous	141 (15)	2.63	2.13–3.25	2.87	2.32–3.56	1.27	1.02–1.57	1.60	1.28–1.99

Model 1: Adjusted for age
Model 2: Adjusted for Model 1 + marital status and education

Supplemental Table S2. Gender-stratified age-adjusted relative risk ratios (RRRs) and their 95% confidence intervals (CIs) for sickness presenteeism days, sickness absence days, and their combinations during a 1-year follow-up among young and early midlife employees of the City of Helsinki, Finland (n = 4,039), by exposure to demanding working conditions in 2017. Multinomial logistic regression.

			No presenteeism days				Presenteeism ≥1 day			
			No sickness absence days (ref.)		Sickness absence ≥1 day		No sickness absence days		Sickness absence ≥1 day	
	Exposure group	n (%)	RRR	CI	RRR	CI	RRR	CI	RRR	CI
Women	*Physically strenuous work*									
	Intermediate	1098 (35)	1.00	–	1.32	0.96–1.82	1.30	0.87–1.93	1.72	1.26–2.36
	Strenuous	1336 (43)	1.00	–	1.23	0.86–1.76	1.32	0.85–2.04	2.52	1.79–3.55
	Mentally strenuous work									
	Intermediate	1983 (64)	1.00	–	1.08	0.80–1.45	1.93	1.29–2.90	2.25	1.67–3.04
	Strenuous	500 (16)	1.00	–	1.15	0.67–1.98	4.31	2.32–8.00	6.95	4.18–11.58
	Time spent in physical work									
	>0–2 hours	1139 (37)	1.00	–	1.24	0.93–1.67	1.30	0.90–1.87	1.45	1.09–1.93
	>2 hours	811 (26)	1.00	–	1.62	1.12–2.34	1.39	0.88–2.17	2.26	1.59–3.21
Men	*Physically strenuous work*									
	Intermediate	457 (48)	1.00	–	1.49	0.94–2.36	1.47	0.84–2.57	1.45	0.94–2.25
	Strenuous	265 (28)	1.00	–	2.09	1.23–3.55	1.36	0.70–2.65	1.93	1.16–3.22
	Mentally strenuous work									
	Intermediate	518 (55)	1.00	–	0.79	0.53–1.18	1.53	0.89–2.63	1.36	0.90–2.04
	Strenuous	131 (15)	1.00	–	1.82	0.81–4.08	5.36	2.20–13.09	6.01	2.80–12.91
	Time spent in physical work									
	>0–2 hours	369 (39)	1.00	–	1.62	1.03–2.53	1.37	0.81–2.31	1.36	0.89–2.08
	>2 hours	273 (29)	1.00	–	2.31	1.41–3.78	1.00	0.53–1.88	1.77	1.10–2.85

Reference categories were physically non-strenuous work, mentally non-strenuous work, and 0 hours spent in physical work.
